# Prognostic Significance of FDG PET/CT in Esophageal Squamous Cell Carcinoma in the Era of the 8th AJCC/UICC Staging System

**DOI:** 10.3389/fonc.2022.861867

**Published:** 2022-06-30

**Authors:** Hyunjong Lee, Kyung Soo Lee, Yang Won Min, Hong Kwan Kim, Jae Ill Zo, Young Mog Shim, Joon Young Choi

**Affiliations:** ^1^Department of Nuclear Medicine, Samsung Medical Center, Sungkyunkwan University School of Medicine, Seoul, South Korea; ^2^Department of Radiology, Samsung Changwon Hospital, Sungkyunkwan University School of Medicine, Seoul, South Korea; ^3^Division of Gastroenterology, Department of Medicine, Samsung Medical Center, Sungkyunkwan University School of Medicine, Seoul, South Korea; ^4^Department of Thoracic Surgery, Samsung Medical Center, Sungkyunkwan University School of Medicine, Seoul, South Korea

**Keywords:** esophageal cancer, squamous cell carcinoma, FDG PET/CT, prognosis, 8th AJCC staging system

## Abstract

**Introduction:**

Recently, the American Joint Committee on Cancer (AJCC)/Union for International Cancer Control (UICC) staging system was updated for its 8th edition. F-18 fluorodeoxyglucose positron emission tomography/computed tomography (FDG PET/CT) is a useful imaging tool to diagnose and predict prognoses for esophageal cancer. However, there was no previous study to explore the role of FDG PET/CT in the staging system based on the 8th edition. The prognostic value of FDG PET/CT was investigated in patients with esophageal squamous cell carcinoma (SqCC) considering the new 8th AJCC/UICC staging system.

**Methods:**

Subjects were 721 patients with esophageal SqCC undergoing pretherapeutic FDG PET/CT. Clinico-pathological variables and the maximum standardized uptake value (SUVmax) of the primary tumor were included in survival analysis. Subgroup analysis was performed to compare hazard ratios according to pathological stage and SUVmax. A new staging classification including FDG uptake was proposed.

**Results:**

In multivariate survival analysis, pathological stage and SUVmax of the primary tumor were selected as independent prognostic factors for overall survival in both the 7th and 8th editions. The proposed new staging system showed better discrimination for overall survival between stage I and II than did the conventional staging system (hazard ratios: 2.250 vs. 1.341).

**Conclusions:**

The FDG uptake of the primary tumor was found to be an independent prognostic factor along with pathological stage based on both 7th and 8th AJCC/UICC staging systems in patients with esophageal SqCC. The suggested new staging system including SUVmax was better for predicting prognoses than the conventional staging system.

## Introduction

Esophageal cancer is a representative disease among malignancies in the digestive system. In 2020, esophageal cancer had the seventh highest incidence and the sixth highest mortality in the world (1). The age-standardized incidence rate of esophageal cancer is highest in Eastern Asia (1). Squamous cell carcinoma (SqCC) is the most common histologic subtype of esophageal cancer, not only in Eastern Asia, but also globally (2). Surgery is the most radical and most common treatment option for esophageal cancer (3). According to stage or physician’s opinion, neoadjuvant concurrent chemoradiotherapy (CCRT) can be performed before surgery (4). After surgery, chemotherapy or radiotherapy can be conducted as adjuvant therapy. Definitive CCRT is another treatment option for patients who cannot be candidates for surgery (5). As most esophageal cancer patients undergo radical operations, it is important to predict the prognosis after surgery.

F-18 fluorodeoxyglucose positron emission tomography/computed tomography (FDG PET/CT) is a robust imaging modality to diagnose and evaluate stages of esophageal cancer (6, 7). It provides additional information of nodal staging and distant metastasis (8). Image findings and parameters of FDG PET/CT are well known to predict the prognosis of esophageal cancer. A high standardized uptake value (SUV) of the primary tumor was a significant risk factor in esophageal cancer based on a meta-analysis (9). Although there have been many studies, they included small numbers of patients or applied meta-analysis designs to overcome the limitations of low subject numbers. Furthermore, there were no previous studies dealing with prognostic values according to all discrete substages (e.g., IA and IB) due to the small numbers of subjects. Therefore, it was not clear whether the FDG PET/CT parameters of the primary tumor were independent prognostic factors over pathological substages or stages.

The tumor–node–metastasis (TNM) cancer staging system suggested by the American Joint Committee on Cancer (AJCC)/Union for International Cancer Control (UICC) is the most used system to evaluate the progression of malignancies including esophageal cancer. In 2017, the 8th edition of the AJCC/UICC staging system was released (10). A remarkable change between the 7th and 8th editions is the subdivision of the T1 stage into T1a and T1b according to invasion depth (11). Staging N3 as IVA and re-defining esophagogastric junction cancer are other changes (11). The classification criteria for the N stage have not been changed. In survival analysis, staging based on the 8th edition of the AJCC/UICC staging system still showed good prognostic stratification performance. However, no previous study has explored the independent prognostic value of FDG PET/CT compared to staging based on the 8th edition of the AJCC/UICC staging system.

In this study, the prognostic value of FDG PET/CT was investigated in a large number of subjects with esophageal SqCC according to the 7th and 8th editions of the AJCC/UICC staging system. The significance of SUVmax as a reference to stratify patients of same pathological stage was also explored.

## Methods

### Subjects

**Figure 1:**
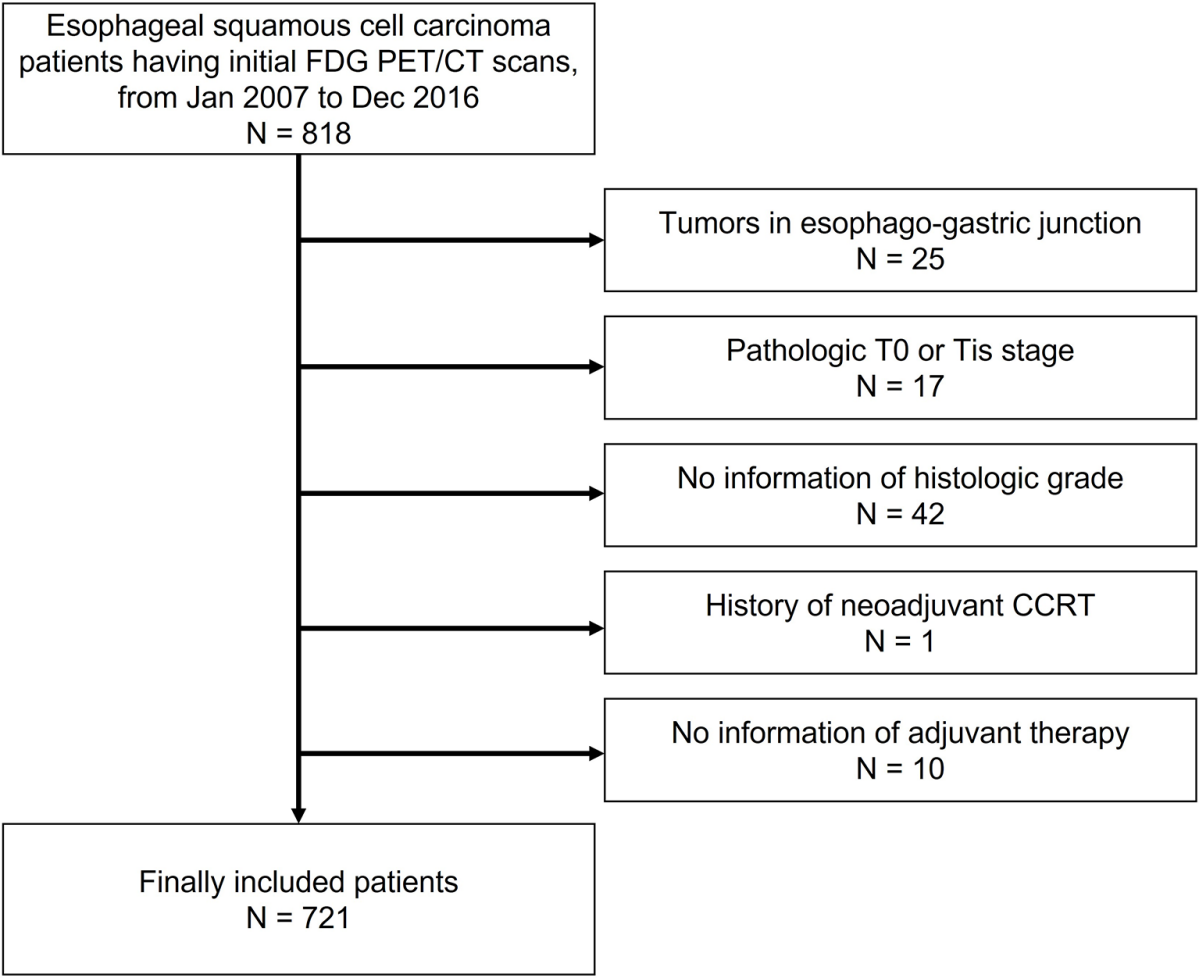
Patient inclusion and exclusion criteria Eight hundred eighteen patients were retrospectively enrolled. Among them, patients with a primary tumor located in the esophago-gastric junction, with pathologic T0 or Tis, without pathologic information of histologic grade, with neo-adjuvant concurrent chemoradiotherapy, or without information of adjuvant therapy were subsequently excluded. Ultimately, 721 patients were included.

Eight hundred eighteen consecutive patients undergoing FDG PET/CT examination for the initial staging of esophageal SqCC and subsequent curative surgery between January 2007 and December 2016 were retrospectively enrolled. Among them, 24 patients with pathology other than SqCC were excluded. Tumors located in the esophago-gastric junction were excluded due to the different staging system and prognosis. Seventeen patients with pathologic T0 or Tis were excluded. Forty-two patients without pathologic information of histologic grade were excluded. Pathologic T stage and histologic grade were diagnosed by pathologists from surgical specimens. Pathologic information was obtained from pathologic reports in electronic medical records. One patient with neo-adjuvant concurrent chemoradiotherapy before FDG PET/CT examination was excluded. Ten patients were excluded due to the absence of information regarding adjuvant therapy after surgery. Therefore, 721 patients were included in this study (**Figure 1**). All patients underwent esophagogastroduodenoscopy (EGD), endoscopic ultrasonography (EUS), and CT of the chest and upper abdomen as a diagnostic workup of esophageal cancer. Our institute review board approved this retrospective cohort study (IRB #2021-06-005), and the informed consent was waived.

### FDG PET/CT Acquisition and Analysis

All patients fasted for at least 6 h and had blood glucose levels of less than 200 mg/dl at the time of their FDG PET/CT scans. Whole-body PET and CT images from basal skull to mid-thigh were acquired 60 min after the injection of 5.0 MBq/kg FDG without intravenous or oral contrast on a Discovery LS or a Discovery STE PET/CT scanner (GE Healthcare, Milwaukee, WI, USA). Continuous spiral CT was performed with an 8-slice helical CT (140 keV, 40–120 mA; Discovery LS) or 16-slice helical CT (140 keV, 30–170 mA; Discovery STE). An emission scan was then obtained from head to thigh for 4 min per frame in 2-dimensional mode (Discovery LS) or 2.5 min per frame in 3-dimensional mode (Discovery STE). PET images were reconstructed using CT for the attenuation correction by the ordered-subsets expectation maximization algorithm with 28 subsets and 2 iterations (matrix 128 × 128, voxel size 4.3 × 4.3 × 3.9 mm; Discovery LS) or ordered-subsets expectation maximization algorithm with 20 subsets and 2 iterations (matrix 128 × 128, voxel size 3.9 × 3.9 × 3.3 mm; Discovery STE).

All images were reviewed by two board-certified nuclear medicine physicians who have over 5 years’ experience, using volume viewer software on a GE Advantage Workstation version 4.7. Maximum SUV (SUVmax) of the primary tumor was measured using a spherical volume of interest over the primary tumor. Lymph nodes were considered metastatic if there was focal prominent FDG uptake compared to normal mediastinal activity matching with a lymph node on CT image without high attenuation and calcification based on the previous studies, which showed a high negative predictive value with a range of 88%–98% (8, 12, 13). The number of FDG PET/CT-positive nodes in each patient was recorded.

### Clinical Variables and Follow-Up

Clinical information including sex, age, performance of adjuvant therapy, and histological grade of the primary tumor was obtained by reviewing electronic medical records. The location of the primary tumor was classified by examination reports of EGD or EUS. Radiologic reports of CT covering the chest and upper abdomen were reviewed, and the numbers of possible metastatic lymph nodes were obtained. Clinical nodal stage (cN stage) was evaluated by the larger numbers of positive lymph nodes for metastasis in FDG PET/CT or CT scans. After we reviewed the pathologic reports, the pathological TNM stage and substage were determined based on both the 7th and 8th editions of the AJCC/UICC staging system.

Adjuvant therapy after surgery was performed according to each patient’s situation and their corresponding physician’s decision. After surgery, all patients were monitored regularly to obtain accurate information regarding recurrence. The follow-up program was every 2–4 months during the first year, every 4–6 months during the next 2 years, and every year thereafter. Every follow-up evaluation included a complete physical examination, complete blood count, biochemical screening, and chest x-ray. CT scans of the chest and upper abdomen were performed from every 6 months to 1 year, or more frequently if clinically indicated. Other tests, including barium contrast esophagography, EGD, and ultrasonography/CT of the neck and abdomen were also performed if clinically indicated.

Recurrence or metastasis was considered when there was an abnormal finding suggesting recurrence or metastasis on serial imaging studies or pathologically confirmed malignancy. The events for survival analysis were defined as recurrence or metastasis and any cause of death. The disease-free and overall survival (OS) durations from the last follow-up or event were recorded for each patient.

### Statistical Analysis

Age as a continuous scale was divided into three groups as a discrete scale according to tertiles for log-rank tests and multivariate analyses. SUVmax as a continuous scale was divided into two groups as a discrete scale according to two cutoff references, the median value of SUVmax and a cutoff value of SUVmax to best discriminate the prognosis of OS in all patients. The latter was explored by the “cox” function in the package “cutoff”. The “cox” function provides hazard ratios (HRs) and *p*-values of Cox regression analysis for each cutoff value. Cutoff values were applied not only for log-rank tests and multivariate analyses but also for suggestion of a reference value for the new staging system. Clinical variables including sex, age with both discrete and continuous scales, location of primary tumor, cN stage, performance of adjuvant therapy, histological grade of primary tumor, pathological T stage based on the 7th edition of the AJCC/UICC staging system (7th pT stage), pathological T stage based on the 8th edition of the AJCC/UICC staging system (8th pT stage), pathological N stage (pN stage), pathological substage based on the 7th edition of the AJCC/UICC staging system (7th pathological substage), pathological substage based on the 8th edition of the AJCC/UICC staging system (8th pathological substage), and SUVmax with both discrete and continuous scales were employed for univariate survival analysis. Both OS and disease-free survival (DFS) were endpoints of analysis. The Cox proportional hazards model was used to evaluate the prognostic power of each variable. HRs and 95% confidence intervals were estimated. Log-rank statistics were also obtained by the Kaplan–Meier method. Significant variables in univariate survival analysis with *p*-values of log-rank statistics lower than 0.05 were included in multivariate survival analysis. Variables with collinearity were excluded.

Patients in each 8th-edition pathological stage (e.g., I and II) were divided into two groups based on the two reference values of SUVmax. HRs and 95% confidence intervals were estimated, and log-rank statistics were also obtained by the Kaplan–Meier method. Subgroups showing no significant difference in HRs were classified into the same group. Kaplan–Meier analysis was conducted in the newly classified groups. All the statistical analyses were performed using R software (v. 4.0.4, R Foundation for Statistical Computing, Vienna, Austria). A *p*-value lower than 0.05 was considered statistically significant.

## Results

### Demographic Data

The clinical characteristics and demographics of the subjects are described in **Table 1**. Overall, 92.5% of patients were male. The median age was 73 years, and the median SUVmax was 4.7. The tumors were located in the middle and lower thoracic esophagus in 90.1% of subjects. CT or FDG PET/CT demonstrated no lymph nodes suspected as metastatic in 484 patients. In pathological findings, 385 patients had no lymph node metastasis. Stage IB was the most common stage in both the 7th pathological substage and the 8th pathological substage. Stage I was the most common in both the 7th pathological stage and the 8th pathological stage.

**Table 1:** Demographic and clinical characteristics of the patients with esophageal squamous cell carcinoma.

Characteristics	Patients, *n* (%)
Sex	
Female	54 (7.5)
Male	667 (92.5)
Age, median (range), years	73 (39–96)
<69	245 (34.0)
69–78	261 (36.2)
≥78	215 (29.8)
SUVmax, median cutoff	4.7
<4.7	362 (50.2)
≥4.7	359 (49.8)
SUVmax, best cutoff	3.4
<3.4	262 (36.3)
≥3.4	459 (63.7)
Location	
Cervical	2 (0.3)
Upper	69 (9.6)
Middle	274 (38.0)
Lower	376 (52.1)
Clinical N stage	
N0	484 (67.1)
N1	182 (25.2)
N2	50 (6.9)
N3	5 (0.7)
Adjuvant therapy	
No	550 (76.3)
Concurrent chemoradiation therapy (CCRT)	6 (0.8)
Radiation therapy (RT)	9 (1.2)
Chemotherapy (CT)	156 (21.6)
Histological grade	
1	83 (11.5)
2	533 (73.9)
3	105 (14.6)
7th pathological T stage	
T1	427 (59.2)
T2	85 (11.8)
T3	200 (27.7)
T4a	4 (0.6)
T4b	5 (0.7)
8th pathological T stage	
T1a	141 (19.6)
T1b	286 (39.7)
T2	85 (11.8)
T3	200 (27.7)
T4a	4 (0.6)
T4b	5 (0.7)
Pathological N stage	
N0	385 (53.4)
N1	185 (25.7)
N2	108 (15.0)
N3	43 (6.0)
7th pathological substage	
IA	33 (4.6)
IB	277 (38.4)
IIA	23 (3.2)
IIB	173 (24.0)
IIIA	107 (14.8)
IIIB	60 (8.3)
IIIC	48 (6.7)
7th pathological stage	
I	310 (43.0)
II	196 (27.2)
III	215 (29.8)
8th pathological substage	
IA	22 (3.1)
IB	287 (39.8)
IIA	47 (6.5)
IIB	126 (17.5)
IIIA	51 (7.1)
IIIB	140 (19.4)
IVA	48 (6.7)
8th pathological stage	
I	309 (42.9)
II	173 (24.0)
III	191 (26.5)
IV	48 (6.7)
Instrument	
Discovery LS	411 (57.0)
Discovery STE	310 (43.0)

### Survival Analysis Data

In univariate survival analysis, sex, age with discrete scale, SUVmax with discrete scale, SUVmax with continuous scale, cN stage, adjuvant therapy, 7th pT stage, 8th pT stage, pN stage, 7th pathological substage, 7th pathological stage, 8th pathological substage, and 8th pathological stage were revealed to be significant prognostic factors for OS (**Table 2**). Sex, SUVmax with discrete scale, SUVmax with continuous scale, cN stage, adjuvant therapy, histological grade, 7th pT stage, 8th pT stage, pN stage, 7th pathological substage, 7th pathological stage, 8th pathological substage, and 8th pathological stage were revealed to be significant prognostic factors for DFS (**Table 2**).

**Table 2:** Univariate Cox regression analysis of survival in esophageal squamous cell carcinoma.

Variable	Categories	Disease-free survival				Overall survival				
		Hazard ratio	95% confidence interval	*p*	*p* of log-rank test		Hazard ratio	95% confidence interval	*p*	*p* of log-rank test	
Sex	Female vs. Male	2.057	1.013–4.178	0.046	0.04		1.894	1.061–3.379	0.031	0.03	
Age	<69				0.4					<0.001	
	69–78	1.175	0.837–1.650	0.351			1.146	0.842–1.560	0.387		
	≥78	0.948	0.651–1.380	0.779			1.905	1.417–2.560	<0.001		
Age (1-year increase)		1.006	0.989–1.022	0.508	0.5		1.035	1.020–1.050	<0.001	<0.001	
Location	Cervical				0.8					0.9	
	Upper	0.499	0.067–3.731	0.498			0.574	0.078–4.240	0.586
	Middle	0.425	0.059–3.065	0.396			0.552	0.077–3.962	0.555
	Lower	0.461	0.064–3.605	0.441			0.590	0.083–4.223	0.600
Clinical N stage	N0				<0.001					<0.001	
	N1	3.084	2.255–4.219	<0.001			1.941	1.494–2.521	<0.001
	N2	4.326	2.739–6.833	<0.001			2.644	1.783–3.922	<0.001
	N3	9.352	2.943–29.716	<0.001			7.279	2.971–17.830	<0.001
Adjuvant therapy	No				<0.001					0.003	
	CCRT	2.866	0.910–9.029	0.072			1.927	0.615–6.034	0.260
	RT	3.627	1.479–8.895	0.005			2.532	1.122–5.714	0.025
	CT	2.219	1.630–3.020	<0.001			1.520	1.160–1.992	0.002
Histological grade	1				0.006					0.07	
	2	0.687	0.450–1.049	0.082			0.869	0.603–1.251	0.449
	3	1.200	0.726–1.982	0.477			1.259	0.809–1.958	0.308
7th pathological T stage	T1				<0.001					<0.001	
	T2	1.519	0.874–2.640	0.138			1.879	1.267–2.787	0.002
	T3	5.996	4.351–8.264	<0.001			4.307	3.319–5.590	<0.001
	T4a	1.940	0.269–14.005	0.511			0.952	0.133–6.824	0.961
	T4b	14.005	4.384–44.741	<0.001			6.683	2.453–18.210	<0.001
8th pathological T stage	T1a				<0.001					<0.001	
	T1b	3.284	1.558–6.979	0.002			1.297	0.838–2.008	0.244
	T2	3.810	1.630–8.902	0.002			2.248	1.358–3.722	0.002
	T3	15.038	7.321–30.888	<0.001			5.157	3.427–7.758	<0.001
	T4a	4.863	0.608–38.888	0.136			1.138	0.155–8.369	0.899
	T4b	35.126	9.607–132.577	<0.001			7.999	2.798–22.868	<0.001
Pathological N stage	N0				<0.001					<0.001	
	N1	2.547	1.699–3.818	<0.001			1.441	1.055–1.969	0.022
	N2	6.652	4.490–9.854	<0.001			3.810	2.803–5.178	<0.001
	N3	14.073	8.913–22.221	<0.001			6.548	4.442–9.652	<0.001
7th pathological substage	IA				<0.001					<0.001	
	IB	1.212	0.368–3.990	0.751			1.360	0.622–2.974	0.441
	IIA	1.788	0.361–8.862	0.477			1.904	0.667–5.436	0.229
	IIB	2.202	0.673–7.204	0.192			1.704	0.773–3.757	0.187
	IIIA	6.920	2.147–22.310	0.001			3.916	1.782–8.606	<0.001
	IIIB	15.233	4.703–49.338	<0.001			9.546	4.304–21.172	<0.001
	IIIC	17.800	5.445–58.195	<0.001			8.852	3.927–19.953	<0.001
8th pathological substage	IA				<0.001					<0.001	
	IB	2.477	0.337–18.190	0.373			1.070	0.431–2.662	0.884
	IIA	4.847	0.606–38.760	0.137			2.131	0.790–5.750	0.135
	IIB	4.557	0.615–33.740	0.138			1.210	0.472–3.102	0.691
	IIIA	8.024	1.060–60.750	0.044			1.746	0.644–4.736	0.274
	IIIB	20.867	2.899–150.200	0.003			5.037	2.047–12.395	<0.001
	IVA	36.052	4.926–263.890	<0.001			7.197	2.821–18.359	<0.001
SUVmax (median cutoff)	<4.7				<0.001					<0.001	
	≥4.7	4.871	3.429–6.919	<0.001			3.232	2.481–4.211	<0.001
SUVmax (best cutoff)	<3.4				<0.001					<0.001	
	≥3.4	6.698	4.117–10.900	<0.001			3.534	2.565–4.869	<0.001
SUVmax (continuous)		1.083	1.066–1.101	<0.001	<0.001		1.069	1.054–1.084	<0.001	<0.001	

**Figure 2:**
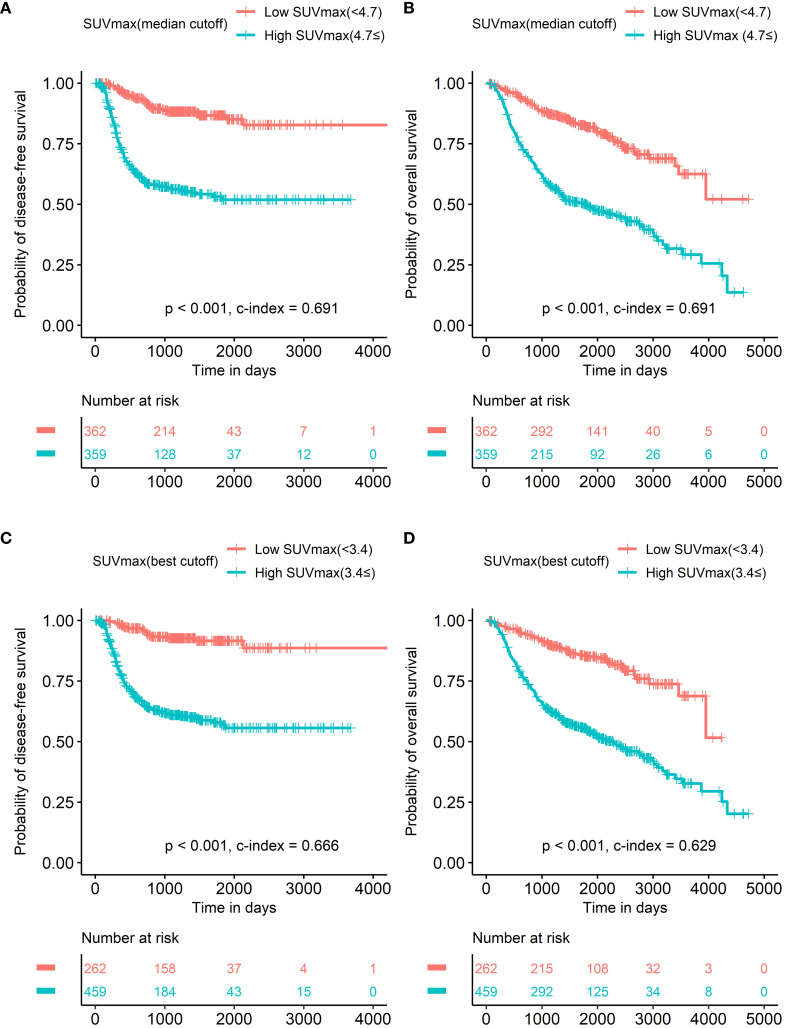
Survival curves according to SUVmax UVmax with median cutoff was a significant prognostic factor in both disease-free survival **(A)** and overall survival **(B)**. SUVmax with the best cutoff to discriminate prognosis of overall survival most accurately in all patients showed the same results in both disease-free survival **(C)** and overall survival **(D)**.

**Figure 3:**
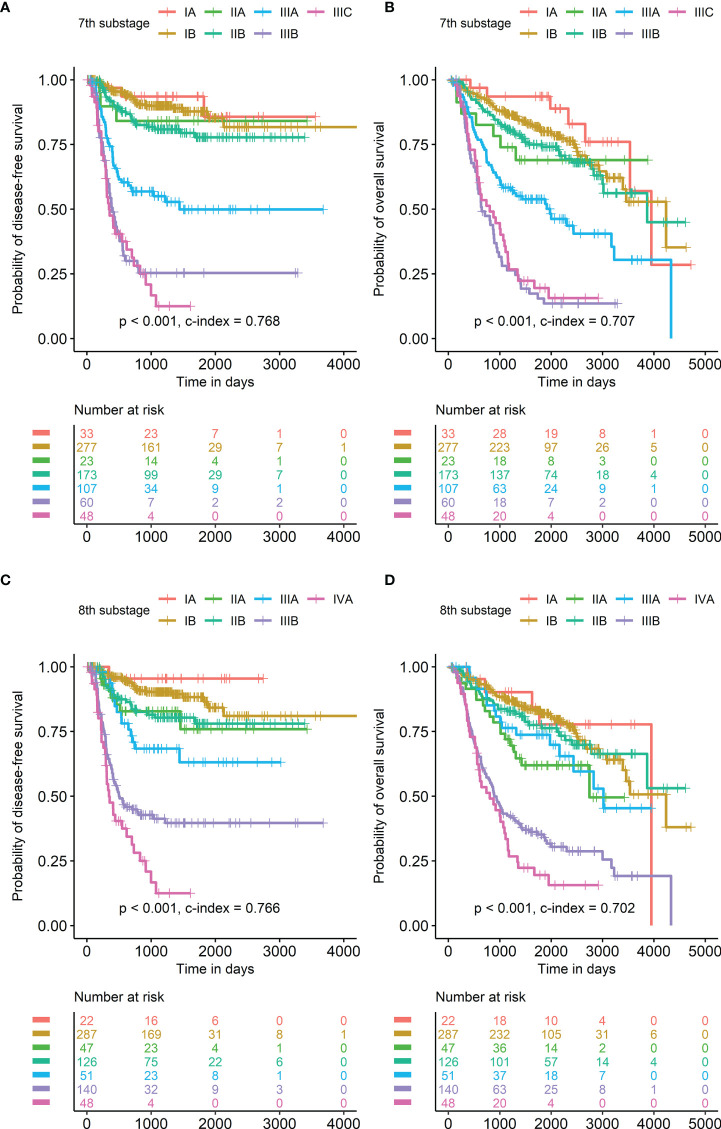
Survival curves according to the AJCC/UICC staging systems Pathological substage based on the 7th edition of the AJCC/UICC staging system was a significant prognostic factor in both disease-free survival **(A)** and overall survival **(B)**. Pathological substage based on the 8th edition of the AJCC/UICC staging system was also a significant prognostic factor in both disease-free survival **(C)** and overall survival **(D)**.

Due to multicollinearity issues, multivariate survival analysis was performed repeatedly according to each PET parameter and staging system (**Tables 3**, **4**). In the multivariate survival analysis, both SUVmax and pathological stage were selected as significant prognostic factors for DFS based on both the 7th- and 8th-edition staging systems. In addition, sex was an independent prognostic factor in the 8th staging system. In multivariate survival analysis, both SUVmax and pathological stage were selected as significant prognostic factors for OS based on both the 7th- and 8th-edition staging systems. The same results were observed irrespective of the SUVmax cutoff method. Survival curves of SUVmax with discrete scale and 7th or 8th pathological substage are displayed in **Figures 2**, **3**.

**Table 3:** Multivariate Cox regression analysis of disease-free survival in esophageal squamous cell carcinoma.

Variable	Categories	7th-edition staging, SUVmax (median cutoff)			7th-edition staging, SUVmax (best cutoff)			8th-edition staging, SUVmax (median cutoff)			8th-edition staging, SUVmax (best cutoff)		
		Hazard ratio	95% confidence interval	*p*	Hazard ratio	95% confidence interval	*p*	Hazard ratio	95% confidence interval	*p*	Hazard ratio	95% confidence interval	*p*
Sex	Female vs. Male	1.989	0.970–4.082	0.061	2.043	0.995–4.194	0.052	2.110	1.030–4.322	0.041	2.153	1.050–4.412	0.036
Clinical N stage	N0												
	N1	1.491	1.063–2.091	0.021	1.456	1.040–2.037	0.029	1.507	1.074–2.116	0.018	1.473	1.052–2.062	0.024
	N2	1.101	0.657–1.844	0.716	1.107	0.664–1.845	0.697	1.291	0.771–2.159	0.331	1.308	0.785–2.178	0.303
	N3	2.222	0.641–7.709	0.208	2.772	0.796–9.652	0.109	2.821	0.829–9.600	0.097	3.410	0.996–11.673	0.051
Adjuvant therapy	No												
	CCRT	0.526	0.159–1.736	0.292	0.563	0.171–1.853	0.344	0.639	0.193–2.119	0.464	0.681	0.206–2.252	0.529
	RT	2.173	0.874–5.403	0.095	2.385	0.959–5.933	0.062	2.348	0.933–5.912	0.070	2.478	0.986–6.228	0.054
	CT	0.742	0.540–1.075	0.121	0.796	0.565–1.122	0.193	0.805	0.569–1.138	0.220	0.842	0.586–1.189	0.328
Histological grade	1												
	2	0.775	0.488–1.231	0.280	0.748	0.471–1.187	0.217	0.737	0.475–1.142	0.172	0.719	0.464–1.113	0.139
	3	0.951	0.553–1.635	0.857	0.971	0.564–1.672	0.916	0.874	0.520–1.469	0.611	0.896	0.533–1.507	0.679
7th pathological stage	IA												
	IB	1.332	0.376–4.720	0.657	1.232	0.347–4.377	0.747						
	IIA	1.131	0.213–5.996	0.885	1.008	0.193–5.272	0.993						
	IIB	1.648	0.452–6.013	0.449	1.387	0.381–5.054	0.620						
	IIIA	4.648	1.278–16.899	0.020	4.101	1.137–14.791	0.031						
	IIIB	9.063	2.472–33.230	<0.001	8.113	2.241–29.368	0.001						
	IIIC	11.541	3.098–42.984	<0.001	9.376	2.539–34.623	<0.001						
8th pathological stage	IA												
	IB							2.730	0.363–20.511	0.329	2.147	0.283–16.272	0.460
	IIA							3.226	0.382–27.272	0.282	2.374	0.279–20.202	0.429
	IIB							3.763	0.486–29.133	0.204	2.688	0.343–21.085	0.347
	IIIA							5.602	0.692–45.356	0.106	3.972	0.487–32.422	0.198
	IIIB							12.610	1.632–97.411	0.015	9.526	1.231–73.718	0.031
	IVA							22.485	2.844–177.804	0.003	15.420	1.936–122.832	0.010
SUVmax (median cutoff)	<4.7												
	≥4.7	2.196	1.426–3.381	<0.001				1.999	1.286–3.106	0.002			
SUVmax (best cutoff)	<3.4												
	≥3.4				3.258	1.886–5.687	<0.001				2.982	1.703–5.219	<0.001

**Table 4:** Multivariate Cox regression analysis of overall survival in esophageal squamous cell carcinoma.

Variable	Categories	7th-edition staging, SUVmax (median cutoff)			7th-edition staging, SUVmax (best cutoff)			8th-edition staging, SUVmax (median cutoff)			8th-edition staging, SUVmax (best cutoff)		
		Hazard ratio	95% confidence interval	*p*	Hazard ratio	95% confidence interval	*p*	Hazard ratio	95% confidence interval	*p*	Hazard ratio	95% confidence interval	*p*
Sex	Female vs. Male	1.626	0.905–2.923	0.104	1.623	0.903–2.914	0.105	1.769	0.986–3.173	0.056	1.784	0.995–3.200	0.052
Age	<69												
	69–78	0.971	0.705–1.338	0.857	0.987	0.717–1.358	0.967	0.939	0.680–1.296	0.702	0.936	0.678–1.291	0.687
	≥78	1.525	1.098–2.118	0.012	1.552	1.120–2.149	0.008	1.559	1.124–2.163	0.008	1.548	1.117–2.146	0.009
Clinical N stage	N0												
	N1	1.097	0.824–1.461	0.525	1.100	0.827–1.462	0.513	1.098	0.823–1.466	0.525	1.091	0.819–1.454	0.550
	N2	0.887	0.571–1.380	0.596	0.916	0.591–1.421	0.696	1.030	0.662–1.601	0.896	1.053	0.679–1.634	0.817
	N3	1.578	0.611–4.075	0.346	1.747	0.675–4.517	0.250	1.835	0.709–4.745	0.211	2.019	0.779–5.233	0.148
Adjuvant therapy	No												
	CCRT	0.576	0.175–1.899	0.365	0.604	0.183–1.989	0.407	0.716	0.217–2.364	0.583	0.734	0.223–2.422	0.612
	RT	1.498	0.647–3.466	0.345	1.635	0.707–3.783	0.251	1.680	0.721–3.913	0.229	1.790	0.770–4.162	0.176
	CT	0.788	0.570–1.089	0.149	0.810	0.586–1.119	0.202	0.871	0.628–1.206	0.405	0.888	0.642–1.230	0.476
7th pathological stage	IA												
	IB	1.346	0.613–2.958	0.459	1.408	0.641–3.106	0.394						
	IIA	1.258	0.424–3.734	0.679	1.385	0.475–4.130	0.551						
	IIB	1.272	0.561–2.884	0.565	1.308	0.582–2.951	0.516						
	IIIA	2.890	1.252–6.671	0.013	3.087	1.354–6.870	0.007						
	IIIB	5.999	2.531–14.217	< 0.001	6.513	2.795–15.246	< 0.001						
	IIIC	6.271	2.561–15.353	< 0.001	6.476	2.690–15.589	< 0.001						
8th pathological stage	IA												
	IB							1.039	0.412–2.622	0.935	0.936	0.368–2.377	0.889
	IIA							1.472	0.512–4.234	0.474	1.354	0.473–3.877	0.572
	IIB							0.972	0.365–2.584	0.954	0.847	0.316–2.271	0.742
	IIIA							1.260	0.438–3.619	0.668	1.101	0.382–3.174	0.859
	IIIB							3.245	1.211–8.697	0.019	2.989	1.125–7.943	0.028
	IVA							4.826	1.718–13.555	0.003	4.216	1.506–11.797	0.006
SUVmax (median cutoff)	<4.7												
	≥4.7	1.937	1.382–2.722	< 0.001				1.718	1.202–2.453	0.003			
SUVmax (best cutoff)	<3.4												
	≥3.4				2.158	1.482–2.892	< 0.001				2.001	1.356–2.953	<0.001

### Proposed New Staging System Including SUVmax

**Figure 4:**
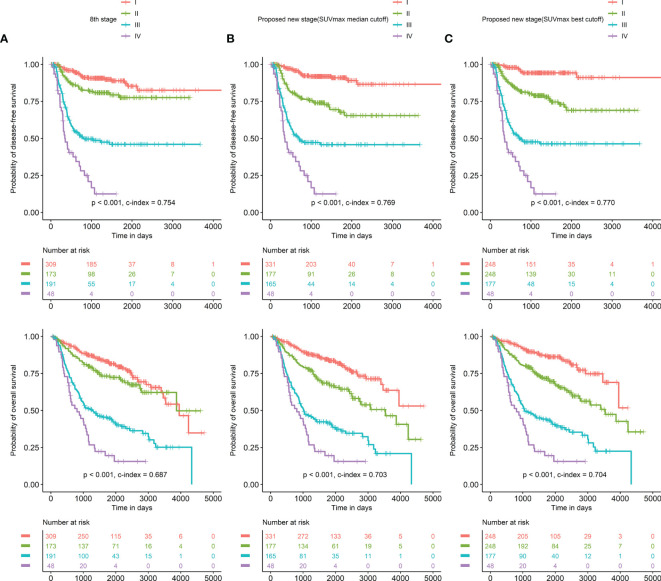
Survival curves according to the conventional stage and the proposed new stage Compared to survival curves based on the 8th-edition pathological stage **(A)**, those based on our proposed new staging system discriminated prognoses better in reference to both SUVmax with median cutoff **(B)** and SUVmax with the best cutoff **(C)**.

**Figure 5:**
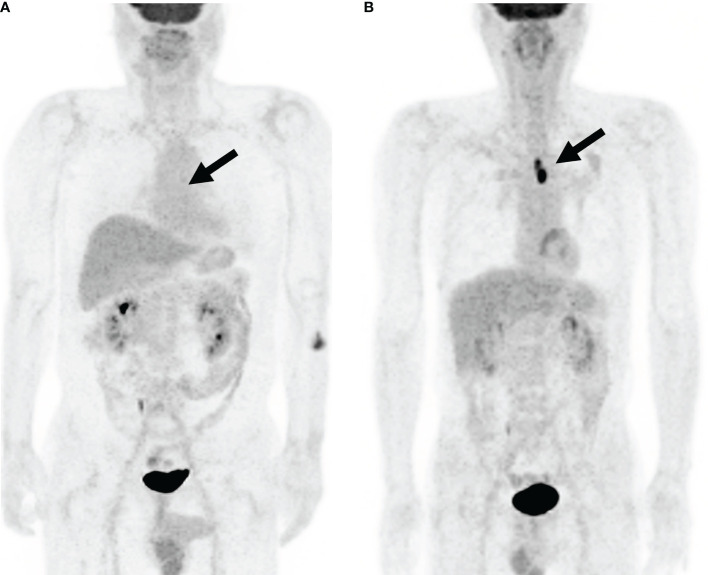
Representative cases of upstaging and downstaging according to SUVmax Maximal intensity projection images of FDG PET/CT demonstrate representative cases of upstaging and downstaging. **(A)** A 73-year-old male patient with ht low SUVmax of 1.0 (red arrow). Thus, he was downstaged into the new proposed stage I. DFS duration was 1,554 days, and OS duration was 2,546 days. **(B)** A 75-year-old male patient with esophageal SqCC of stage I. The tumor was clearly demonstrated in the image due to its high SUVmax of 16.0 (red arrow). Thus, he was upstaged into the new proposed stage II (**Figure 5b**). DFS duration was 329 days, and OS duration was 496 days.

Subgroup analysis was performed to explore the role of SUVmax in each stage and to adjust the staging system to discriminate prognoses better. HRs for OS were calculated in each subgroup classified according to each 8th pathological stage and SUVmax group (**Supplementary** **Table 1**). The HRs of stage I subjects with low SUVmax and stage II subjects with low SUVmax demonstrated no significance differences from each other. Likewise, the HRs of stage I subjects with high SUVmax, stage II subjects with high SUVmax, and stage III subjects with low SUVmax demonstrated no significance difference from each other. Our proposed new staging system was designed based on these results. Stage II subjects with low SUVmax were downstaged into a new stage I. Stage I subjects with high SUVmax were upstaged into a new stage II. Stage III subjects with low SUVmax were downstaged into a new stage II. In the conventional staging system, the HRs of stage I subjects and stage II subjects showed no significant difference. In contrast, the HRs of stage I subjects and stage II subjects showed significant differences in the proposed new staging system (**Table 5**). Compared to survival curves based on the 8th pathological stage, those based on the improved staging system discriminated prognosis better (**Figure 4**). The same results were observed irrespective of the SUVmax cutoff method. **Figure 5** demonstrates two representative cases upstaged or downstaged according to the SUVmax of the primary tumor.

**Table 5:** Univariate Cox regression analysis of overall survival according to the conventional stage and the proposed new stage including SUVmax in esophageal squamous cell carcinoma.

Variable	Categories	Hazard ratio	95% confidence interval	*p*
8th pathological stage	I	1.000		
	II	1.341	0.934–1.926	0.112
	III	3.681	2.728–4.968	<0.001
	IV	6.687	4.478–9.986	<0.001
Proposed new stage (SUVmax, median cutoff)	I (I with low SUVmax + II with low SUVmax)	1.000		
	II (I with high SUVmax + II with high SUVmax + III with low SUVmax)	2.010	1.422–2.840	<0.001
	III (III with high SUVmax)	4.805	3.517–6.564	<0.001
	IV	7.837	5.220–11.767	<0.001
Proposed new stage (SUVmax, best cutoff)	I (I with low SUVmax + II with low SUVmax)	1.000		
	II (I with high SUVmax + II with high SUVmax + III with low SUVmax)	2.250	1.544–3.279	<0.001
	III (III with high SUVmax)	5.474	3.806–7.872	<0.001
	IV	9.473	6.048–14.839	<0.001

## Discussion

The present study showed that the SUVmax of the primary tumor on FDG PET/CT was a significant prognostic factor on both continuous and discrete scales in univariate analysis. In addition, it was selected as an independent prognostic factor as well as a pathological TNM substage/stage based on both the 7th and 8th editions of the AJCC/UICC staging system in multivariate analysis. When subjects were grouped into our proposed new staging system including the 8th pathological stage and SUVmax, the new staging system was revealed to discriminate prognosis better than the conventional pathological staging system.

SUV is a semi-quantitative value for FDG PET/CT, reflecting the glucose metabolism of tissue. It is very useful to diagnose malignancy, evaluate aggressiveness, and predict the prognosis of cancer (14). Also, it is utilized in many radiogenomics approaches to investigate tumor biology non-invasively (15). There are other quantitative parameters derived from FDG PET/CT, such as metabolic tumor volume (MTV) and total lesion glycolysis (TLG). There have been several reports that have suggested the prognostic roles of MTV and TLG from pre-treatment FDG PET/CT in esophageal cancer (16–19). However, MTV and TLG have lesser reproducibility than SUVmax in terms of various and arbitrary tumor segmentation methods. Furthermore, an additional process is needed to delineate tumors and calculate MTV and TLG so that they are difficult to utilize in clinical fields. Compared to other volumetric parameters, SUVmax is the most common parameter in terms of its high reproducibility and simplicity and is easily clinically applicable in routine practice.

In esophageal cancer, the usefulness of FDG PET/CT was investigated in many previous reports (20, 21). Among them, a few studies showed that SUV was an independent predictor for OS after surgery (22–25). However, most of the previous studies recruited less than 100 subjects; Cerfolio et al. employed 89 patients (22), Cheze-Le Rest et al. used 47 patients (23), and Sepesi et al. utilized 72 patients (24). Kato et al. enrolled 184 patients and analyzed the prognostic role of peak SUV with TNM stage (e.g., I and II) based on the 6th edition of the AJCC/UICC staging system (25). Other scholars performed meta-analyses to include larger numbers of patients. Pan et al. incorporated 542 patients from 10 studies (9). However, the study included heterogeneous histologic subtypes and a threshold definition of SUV. Han et al. employed 1,294 patients from 16 studies to explore the prognostic value of FDG PET/CT parameters (16). However, CCRT without operation was performed in the majority of study subjects. To the best of our knowledge, this is the first study to explore the prognostic power of FDG PET/CT with a large number of subjects from a single institute. It is significant in that all patients were diagnosed with SqCC and underwent radical operations. Therefore, this study strongly suggests that SUVmax has a prognostic role in patients with esophageal SqCC undergoing curative surgery.

In this study, seven discrete substages (e.g., IA and IB) were used in univariate and multivariate Cox regression analyses. Notably, the prognostic power of SUV was significant with substage/stage not only based on the 7th edition of the AJCC/UICC staging system but also based on the 8th edition. In the 8th edition, it is remarkable that the T1 stage was classified more precisely according to the invasion depth of a tumor and that N3 was classified into stage IVA. One institute demonstrated that the staging system based on the 8th edition of the AJCC TNM staging system was superior to that based on the 7th edition in the prognosis of OS (26). Despite the improved prognostic power of the 8th-edition staging, this study showed that SUVmax still has its prognostic role. The present study may be the first study to investigate the prognostic role of FDG PET/CT based on both the 7th- and 8th-edition staging systems including all substages.

Herein, a new staging system including the 8th-edition staging system and the SUVmax of the primary tumor was proposed. The proposed new staging system demonstrated better survival curves than the conventional staging system. Notably, stage II and stage III patients with low SUVmax could be downstaged into the new stage I and stage II, respectively, in terms of prognostic stratification. Stage I patients with high SUVmax could be upstaged into the new stage II. This study suggests that SUVmax of the primary tumor deserves to be included in the next edition of staging system for esophageal SqCC to discriminate prognosis more accurately. Specifically, 3.4 or 4.7 is suggested as a reference value to upstage or downstage patients. In contrast, subjects with stage IV were not adjusted because there was no prognostic difference regardless of SUVmax reference. This indicates that FDG PET/CT may have a better prognostic role in the early stages of esophageal SqCC. In the current staging system, patients without lymph node metastasis (N0) are classified into an early stage below stage II. Therefore, it is presumed that biologic characteristics of the primary tumor affect prognosis more significantly in early stages than in advanced stages.

In the clinical field, the conventional TNM stage is commonly used to select appropriate treatment options and predict prognoses. The National Comprehensive Cancer Network guideline recommends esophagectomy as a primary treatment option for patients with low-risk stage II esophageal SqCC, as compared to preoperative or definitive chemoradiation for those with high-risk stage II esophageal SqCC (27). The guideline suggests the presence of lymphovascular invasion, size of tumor, and grade of differentiation as criteria for risk classification. Based on the results of this study, we suggest the SUVmax of the primary tumor as another favorable criterion to select high-risk stage II patients in terms of its good prognostic prediction power, the non-invasiveness of FDG PET/CT examination, and the simplicity of SUVmax. After radical operation, the surveillance follow-up duration is recommended as 3–6 months for 1–2 years. The result of this study implies that follow-up intervals for those with high FDG uptake should be shorter than the intervals for those with low FDG uptake considering the high risk for DFS and OS. In brief, the present study suggests that patients with the same TNM stage can be treated with different treatment options and follow-up plans according to the FDG uptake of primary tumor. Further study is warranted to evaluate the role of FDG PET/CT in selecting an appropriate treatment option in esophageal SqCC patients, especially those in stage II.

This study has several limitations. First, two different kinds of PET/CT scanners were used in this study, which might affect the measurement of SUVmax. Nevertheless, one study showed that SUVs from different instruments were not significantly different in a phantom study (28). Therefore, the analysis was performed based on the hypothesis that SUVs from different instruments can be analyzed without a specific integration process. Furthermore, subgroup analysis according to the PET/CT scanner model showed that SUVmax had a similar prognostic power irrespective of the PET/CT scanner model (**Supplementary Tables 2–7**). Second, only subjects undergoing curative surgery as initial treatment were included in this study for accurate pathological staging. Therefore, relatively small numbers of subjects with advanced stages were included. Further study including all esophageal cancer patients treated with various kinds of therapy is warranted. Third, our proposed new staging system was designed only for pathological stage, not for pathological substage. There was a lack of subjects in specific substages for subgroup analysis to compare HRs according to SUVmax. Further study with more patients could substantiate the value of SUVmax for upstaging or downstaging in pathological substages. Finally, due to the retrospective study design, subjects who underwent therapy based on the 6th- or 7th-edition staging system were also included. A further prospective study including subjects undergoing appropriate treatment according to the 8th-edition staging system is necessary.

In conclusion, the FDG uptake of the primary tumor on FDG PET/CT was found to be an independent prognostic factor along with pathological stage based on both 7th- and 8th-edition staging systems in patients with esophageal SqCC undergoing curative surgery. The proposed new staging system including SUVmax may be better for predicting prognoses than the conventional staging system.

## Data Availability Statement

The original contributions presented in the study are included in the article/**Supplementary Material**. Further inquiries can be directed to the corresponding author.

## Ethics Statement

The studies involving human participants were reviewed and approved by the Samsung Medical Center Institutional Review Board. Written informed consent for participation was not required for this study in accordance with the national legislation and the institutional requirements.

## Author Contributions

HL and JC designed the study. YM, HK, JZ, and YS contributed to data collection. HL and JC performed data analysis and interpretation. HL drafted the article. KL and JC provided critical revision of the article. All authors contributed to the article and approved the submitted version.

## Funding

This work was supported by the National R & D Program for Cancer Control of the Ministry of Health and Welfare of Korea (#1720180) and Future Medicine 20*30 Project of the Samsung Medical Center (#SMO1220071).

## Conflict of Interest

The authors declare that the research was conducted in the absence of any commercial or financial relationships that could be construed as a potential conflict of interest.

## Publisher’s Note

All claims expressed in this article are solely those of the authors and do not necessarily represent those of their affiliated organizations, or those of the publisher, the editors and the reviewers. Any product that may be evaluated in this article, or claim that may be made by its manufacturer, is not guaranteed or endorsed by the publisher.
