# Corrigendum: Prognostic significance of FDG PET/CT in esophageal squamous cell carcinoma in the era of the 8th AJCC/UICC staging system

**DOI:** 10.3389/fonc.2024.1505996

**Published:** 2024-10-28

**Authors:** Hyunjong Lee, Kyung Soo Lee, Yang Won Min, Hong Kwan Kim, Jae Ill Zo, Young Mog Shim, Joon Young Choi

**Affiliations:** ^1^ Department of Nuclear Medicine, Samsung Medical Center, Sungkyunkwan University School of Medicine, Seoul, Republic of Korea; ^2^ Department of Radiology, Samsung Changwon Hospital, Sungkyunkwan University School of Medicine, Seoul, Republic of Korea; ^3^ Division of Gastroenterology, Department of Medicine, Samsung Medical Center, Sungkyunkwan University School of Medicine, Seoul, Republic of Korea; ^4^ Department of Thoracic Surgery, Samsung Medical Center, Sungkyunkwan University School of Medicine, Seoul, Republic of Korea

**Keywords:** esophageal cancer, squamous cell carcinoma, FDG PET/CT, prognosis, 8th AJCC staging system

In the published article, there was an error in **Figure 2** as published. The figure legends of all four panels was reversed. The corrected **Figure 2** and its caption appear below.

**Figure 2:**
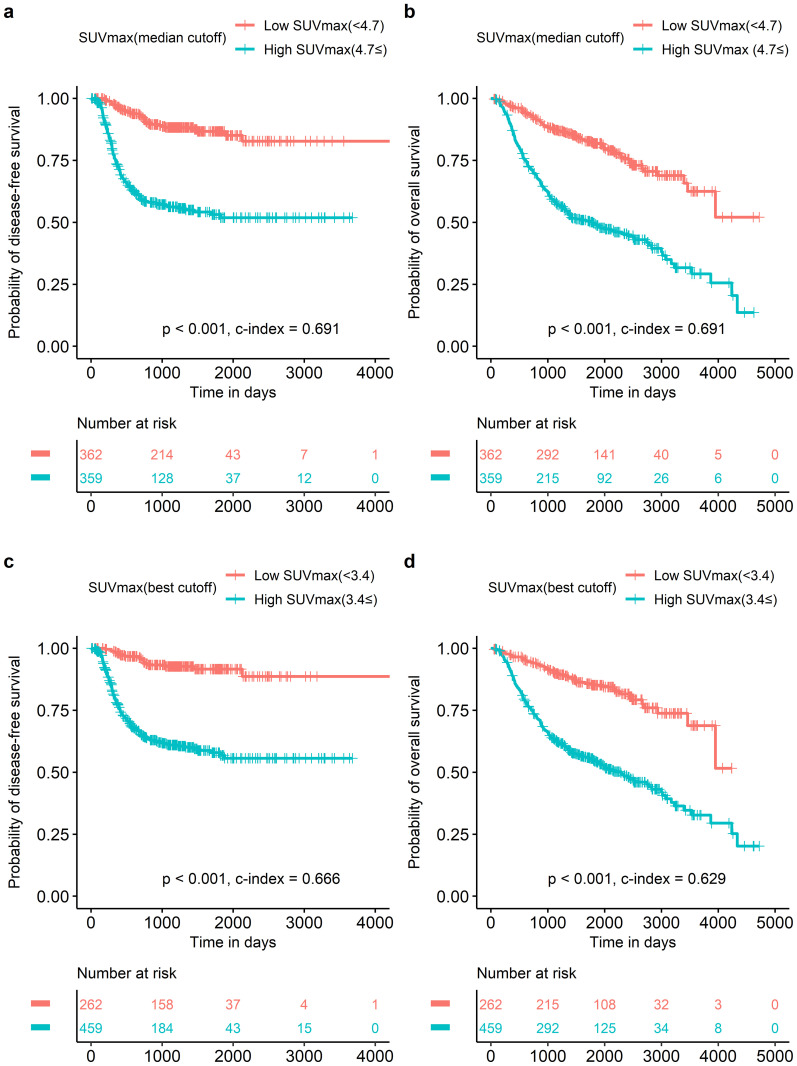
Survival curves according to SUVmax UVmax with median cutoff was a significant prognostic factor in both disease-free survival **(A)** and overall survival **(B)**. SUVmax with the best cutoff to discriminate prognosis of overall survival most accurately in all patients showed the same results in both disease-free survival **(C)** and overall survival **(D)**.

The authors apologize for this error and state that this does not change the scientific conclusions of the article in any way. The original article has been updated.

