# Supplementary material for: Prognostic Significance of FDG PET/CT in Esophageal Squamous Cell Carcinoma in the Era of the 8th AJCC/UICC Staging System
Source: Front Oncol. 2022 Jun 30;12:861867. doi: 10.3389/fonc.2022.861867 (PMC9280981; doi:10.3389/fonc.2022.861867)
Supplement: Supplementary file 1 [file Table_1.docx]

**SUPPLEMENTARY TABLES**

**Supplementary Table 1. Hazard ratios of overall survival in each subgroup according to each 8^th^ pathological stage and SUVmax**

| **Pathological stage** | **SUVmax (median cutoff)** | | **SUVmax (best cutoff)** | |
| --- | --- | --- | --- | --- |
|  | **Low SUVmax (<4.7) HR (n)** | **High SUVmax (4.7≤)**  **HR (n)** | **Low SUVmax (<3.4) HR (n)** | **High SUVmax (3.4≤)**  **HR (n)** |
| I | 1.000 (261) | 1.883 (48) | 1.000 (204) | 2.327 (105) |
| II | 0.893 (70) | 2.021 (103) | 1.108 (44) | 2.243 (129) |
| III | 1.887 (26) | 4.691 (165) | 2.542 (14) | 5.583 (177) |
| IV | 7.837 (5) | 7.634 (43) | - (0) | 9.661 (48) |

**Supplementary Table 2. Univariate Cox regression analysis of survival in subjects with ^18^F-FDG PET/CT using discovery LS**

| **Variable** | **Categories** | **Disease-free survival** | | | | **Overall survival** | | | | |  |
| --- | --- | --- | --- | --- | --- | --- | --- | --- | --- | --- | --- |
|  |  | **Hazard ratio** | **95% confidence interval** | **P** | **P of log-rank test** | | **Hazard ratio** | **95% confidence interval** | **P** | **P of log-rank test** | |
| Sex | Female vs. Male | 1.832 | 0.853-3.937 | 0.121 | 0.1 | | 1.778 | 0.938-3.370 | 0.078 | 0.07 | |
| Age | <69 |  |  |  | 0.5 | |  |  |  | 0.02 | |
|  | 69~78 | 1.072 | 0.699-1.644 | 0.750 |  |  | 1.109 | 0.757-1.625 | 0.595 |  |  |
|  | 78≤ | 0.815 | 0.503-1.322 | 0.408 |  |  | 1.618 | 1.110-2.358 | 0.012 |  |  |
| Age  (1-yr increase) |  | 0.999 | 0.977-1.021 | 0.903 | 0.9 | | 1.030 | 1.011-1.049 | 0.002 | 0.002 | |
| Location | Upper |  |  |  | 0.5 | |  |  |  | 0.5 | |
|  | Middle | 0.705 | 0.378-1.315 | 0.272 |  |  | 0.832 | 0.487-1.422 | 0.501 |  |  |
|  | Lower | 0.829 | 0.455-1.510 | 0.540 |  |  | 0.989 | 0.589-1.622 | 0.968 |  |  |
| Clinical N stage | N0 |  |  |  | < 0.001 | |  |  |  | < 0.001 | |
|  | N1 | 2.743 | 1.839-4.094 | < 0.001 |  |  | 1.799 | 1.284-2.522 | < 0.001 |  |  |
|  | N2 | 3.989 | 2.277-6.987 | < 0.001 |  |  | 2.720 | 1.689-4.380 | < 0.001 |  |  |
|  | N3 | 3.164 | 0.437-22.920 | 0.254 |  |  | 6.181 | 1.948-19.615 | 0.002 |  |  |
| Adjuvant therapy | No |  |  |  | < 0.001 | |  |  |  | 0.02 | |
|  | CCRT | 2.795 | 0.684-11.422 | 0.152 |  |  | 1.713 | 0.423-6.940 | 0.451 |  |  |
|  | RT | 4.307 | 1.730-10.723 | 0.002 |  |  | 2.732 | 1.200-6.216 | 0.017 |  |  |
|  | CT | 2.462 | 1.664-3.644 | < 0.001 |  |  | 1.451 | 1.026-2.053 | 0.035 |  |  |
| Histological grade | 1 |  |  |  | 0.01 | |  |  |  | 0.009 | |
|  | 2 | 0.961 | 0.524-1.764 | 0.899 |  |  | 1.015 | 0.626-1.646 | 0.953 |  |  |
|  | 3 | 1.933 | 0.956-3.910 | 0.067 |  |  | 1.882 | 1.052-3.367 | 0.033 |  |  |
| 7^th^ pathological T stage | T1 |  |  |  | < 0.001 | |  |  |  | < 0.001 | |
|  | T2 | 1.388 | 0.641-3.005 | 0.405 |  |  | 1.585 | 0.923-2.724 | 0.095 |  |  |
|  | T3 | 5.862 | 3.865-8.891 | < 0.001 |  |  | 3.923 | 2.818-5.461 | < 0.001 |  |  |
|  | T4a | 1.852 | 0.253-13.543 | 0.544 |  |  | 0.853 | 0.118-6.162 | 0.875 |  |  |
|  | T4b | 36.073 | 8.468-153.669 | < 0.001 |  |  | 13.368 | 4.114-43.442 | < 0.001 |  |  |
| 8^th^ pathological T stage | T1a |  |  |  | < 0.001 | |  |  |  | < 0.001 | |
|  | T1b | 2.692 | 1.039-6.971 | 0.041 |  |  | 1.257 | 0.707-2.235 | 0.436 |  |  |
|  | T2 | 2.974 | 0.973-9.091 | 0.056 |  |  | 1.861 | 0.939-3.688 | 0.075 |  |  |
|  | T3 | 12.556 | 5.062-31.145 | < 0.001 |  |  | 4.607 | 2.703-7.852 | < 0.001 |  |  |
|  | T4a | 3.968 | 0.464-33.974 | 0.208 |  |  | 1.001 | 0.133-7.552 | 0.999 |  |  |
|  | T4b | 77.266 | 14.707-405.928 | < 0.001 |  |  | 15.698 | 4.495-54.816 | < 0.001 |  |  |
| Pathological N stage | N0 |  |  |  | < 0.001 | |  |  |  | < 0.001 | |
|  | N1 | 1.917 | 1.130-3.252 | 0.016 |  |  | 1.165 | 0.779-1.742 | 0.457 |  |  |
|  | N2 | 6.403 | 3.872-10.589 | < 0.001 |  |  | 3.375 | 2.271-5.015 | < 0.001 |  |  |
|  | N3 | 12.679 | 7.305-22.007 | < 0.001 |  |  | 6.042 | 3.829-9.533 | < 0.001 |  |  |
| 7^th^ pathological substage | IB |  |  |  | < 0.001 | |  |  |  | < 0.001 | |
|  | IIA | 1.763 | 0.521-5.960 | 0.362 |  |  | 1.650 | 0.740-3.681 | 0.221 |  |  |
|  | IIB | 1.184 | 0.608-2.303 | 0.620 |  |  | 1.005 | 0.627-1.610 | 0.983 |  |  |
|  | IIIA | 3.757 | 2.045-6.902 | < 0.001 |  |  | 2.078 | 1.290-3.348 | 0.003 |  |  |
|  | IIIB | 11.720 | 6.471-21.227 | < 0.001 |  |  | 5.902 | 3.694-9.429 | < 0.001 |  |  |
|  | IIIC | 12.046 | 6.607-21.960 | < 0.001 |  |  | 5.694 | 3.526-9.193 | < 0.001 |  |  |
| 8^th^ pathological substage | IA |  |  |  | < 0.001 | |  |  |  | < 0.001 | |
|  | IB | 1.918 | 0.255-14.420 | 0.527 |  |  | 1.072 | 0.382-3.007 | 0.895 |  |  |
|  | IIA | 3.181 | 0.355-28.470 | 0.301 |  |  | 2.343 | 0.754-7.283 | 0.141 |  |  |
|  | IIB | 2.703 | 0.354-20.660 | 0.338 |  |  | 1.083 | 0.371-3.158 | 0.884 |  |  |
|  | IIIA | 5.356 | 0.670-42.830 | 0.114 |  |  | 1.341 | 0.403-4.462 | 0.632 |  |  |
|  | IIIB | 13.779 | 1.897-100.060 | 0.010 |  |  | 4.211 | 1.524-11.640 | 0.006 |  |  |
|  | IVA | 25.859 | 3.497-191.250 | 0.001 |  |  | 6.880 | 2.406-19.672 | < 0.001 |  |  |
| SUVmax (median cutoff) | <4.7 |  |  |  | < 0.001 | |  |  |  | < 0.001 | |
|  | 4.7≤ | 4.057 | 2.602-6.327 | < 0.001 |  |  | 2.651 | 1.899-3.703 | < 0.001 |  |  |
| SUVmax (best cutoff) | <3.4 |  |  |  | < 0.001 | |  |  |  | <0.001 | |
|  | 3.4≤ | 5.976 | 3.122-11.440 | < 0.001 |  |  | 2.786 | 1.856-4.180 | <0.001 |  |  |
| SUVmax (continuous) |  | 1.068 | 1.046-1.090 | < 0.001 | < 0.001 | | 1.061 | 1.042-1.080 | < 0.001 | < 0.001 | |

**Supplementary Table 3. Multivariate Cox regression analysis of disease-free survival in subjects with ^18^F-FDG PET/CT using discovery LS**

| **Variable** | **Categories** | **7^th^ staging,  SUVmax (median cutoff)** | | | **7^th^ staging,  SUVmax (best cutoff)** | | | **8^th^ staging,  SUVmax (median cutoff)** | | | **8^th^ staging,  SUVmax (best cutoff)** | | |
| --- | --- | --- | --- | --- | --- | --- | --- | --- | --- | --- | --- | --- | --- |
|  |  | **Hazard ratio** | **95% confidence interval** | **P** | **Hazard ratio** | **95% confidence interval** | **P** | **Hazard ratio** | **95% confidence interval** | **P** | **Hazard ratio** | **95% confidence interval** | **P** |
| Clinical  N stage | N0 |  |  |  |  |  |  |  |  |  |  |  |  |
|  | N1 | 1.350 | 0.867-2.102 | 0.184 | 1.324 | 0.852-2.058 | 0.212 | 1.332 | 0.857-2.071 | 0.203 | 1.317 | 0.851-2.040 | 0.217 |
|  | N2 | 1.104 | 0.585-2.084 | 0.761 | 1.136 | 0.606-2.129 | 0.692 | 1.338 | 0.710-2.518 | 0.368 | 1.363 | 0.728-2.552 | 0.333 |
|  | N3 | 0.498 | 0.062-4.009 | 0.512 | 0.627 | 0.077-5.125 | 0.663 | 0.800 | 0.102-6.264 | 0.831 | 0.962 | 0.122-7.602 | 0.971 |
| Adjuvant therapy | No |  |  |  |  |  |  |  |  |  |  |  |  |
|  | CCRT | 0.461 | 0.103-2.060 | 0.310 | 0.510 | 0.115-2.272 | 0.377 | 0.578 | 0.129-2.591 | 0.474 | 0.634 | 0.142-2.828 | 0.550 |
|  | RT | 2.949 | 1.152-7.550 | 0.024 | 3.359 | 1.306-8.636 | 0.012 | 3.074 | 1.135-8.330 | 0.027 | 3.401 | 1.273-9.083 | 0.015 |
|  | CT | 0.721 | 0.606-1.496 | 0.833 | 1.013 | 0.644-1.592 | 0.957 | 1.006 | 0.648-1.560 | 0.980 | 1.072 | 0.691-1.664 | 0.756 |
| Histological grade | 1 |  |  |  |  |  |  |  |  |  |  |  |  |
|  | 2 | 0.721 | 0.377-1.379 | 0.323 | 0.719 | 0.377-1.373 | 0.318 | 0.685 | 0.357-1.314 | 0.255 | 0.696 | 0.363-1.334 | 0.275 |
|  | 3 | 1.066 | 0.507-2.238 | 0.867 | 1.168 | 0.556-2.451 | 0.682 | 0.982 | 0.465-2.071 | 0.961 | 1.081 | 0.513-2.279 | 0.837 |
| 7^th^ pathological stage | IB |  |  |  |  |  |  |  |  |  |  |  |  |
|  | IIA | 1.103 | 0.309-3.945 | 0.880 | 1.084 | 0.309-3.808 | 0.900 |  |  |  |  |  |  |
|  | IIB | 0.855 | 0.419-1.744 | 0.666 | 0.782 | 0.383-1.595 | 0.499 |  |  |  |  |  |  |
|  | IIIA | 2.321 | 1.135-4.745 | 0.021 | 2.170 | 1.073-4.386 | 0.031 |  |  |  |  |  |  |
|  | IIIB | 7.854 | 3.759-16.407 | < 0.001 | 7.200 | 3.485-14.875 | < 0.001 |  |  |  |  |  |  |
|  | IIIC | 8.251 | 3.986-17.080 | < 0.001 | 6.961 | 3.362-14.415 | < 0.001 |  |  |  |  |  |  |
| 8th pathological stage | IA |  |  |  |  |  |  |  |  |  |  |  |  |
|  | IB |  |  |  |  |  |  | 1.032 | 0.121-8.803 | 0.977 | 0.954 | 0.115-7.946 | 0.965 |
|  | IIA |  |  |  |  |  |  | 0.974 | 0.090-10.586 | 0.983 | 0.851 | 0.082-8.865 | 0.893 |
|  | IIB |  |  |  |  |  |  | 0.992 | 0.113-8.689 | 0.995 | 0.820 | 0.095-7.049 | 0.857 |
|  | IIIA |  |  |  |  |  |  | 1.806 | 0.192-16.957 | 0.605 | 1.405 | 0.153-12.912 | 0.764 |
|  | IIIB |  |  |  |  |  |  | 3.949 | 0.455-34.285 | 0.213 | 3.362 | 0.403-28.059 | 0.263 |
|  | IVA |  |  |  |  |  |  | 8.070 | 0.916-71.104 | 0.060 | 6.157 | 0.714-53.061 | 0.098 |
| SUVmax (median cutoff) | <4.7 |  |  |  |  |  |  |  |  |  |  |  |  |
|  | 4.7≤ | 1.809 | 1.064-3.074 | 0.029 |  |  |  | 1.688 | 0.981-2.907 | 0.059 |  |  |  |
| SUVmax (best cutoff) | <3.4 |  |  |  |  |  |  |  |  |  |  |  |  |
|  | 3.4≤ |  |  |  | 2.548 | 1.206-5.383 | 0.014 |  |  |  | 2.532 | 1.203-5.330 | 0.014 |

**Supplementary Table 4. Multivariate Cox regression analysis of overall survival in subjects with ^18^F-FDG PET/CT using discovery LS**

| **Variable** | **Categories** | **7^th^ staging,  SUVmax (median cutoff)** | | | **7^th^ staging,  SUVmax (best cutoff)** | | | **8^th^ staging,  SUVmax (median cutoff)** | | | **8^th^ staging,  SUVmax (best cutoff)** | | |
| --- | --- | --- | --- | --- | --- | --- | --- | --- | --- | --- | --- | --- | --- |
|  |  | **Hazard ratio** | **95% confidence interval** | **P** | **Hazard ratio** | **95% confidence interval** | **P** | **Hazard ratio** | **95% confidence interval** | **P** | **Hazard ratio** | **95% confidence interval** | **P** |
| Age | <69 |  |  |  |  |  |  |  |  |  |  |  |  |
|  | 69~78 | 0.875 | 0.579-1.323 | 0.527 | 0.917 | 0.609-1.380 | 0.678 | 0.859 | 0.569-1.298 | 0.471 | 0.888 | 0.588-1.341 | 0.572 |
|  | 78≤ | 1.244 | 0.823-1.879 | 0.300 | 1.277 | 0.848-1.922 | 0.242 | 1.310 | 0.865-1.982 | 0.202 | 1.327 | 0.878-2.006 | 0.179 |
| Clinical  N stage | N0 |  |  |  |  |  |  |  |  |  |  |  |  |
|  | N1 | 1.094 | 0.749-1.598 | 0.642 | 1.100 | 0.755-1.602 | 0.621 | 1.072 | 0.732-1.571 | 0.720 | 1.075 | 0.736-1.570 | 0.708 |
|  | N2 | 1.042 | 0.597-1.818 | 0.885 | 1.067 | 0.614-1.853 | 0.818 | 1.273 | 0.728-2.227 | 0.398 | 1.291 | 0.741-2.249 | 0.367 |
|  | N3 | 1.260 | 0.389-4.553 | 0.724 | 1.316 | 0.361-4.804 | 0.678 | 1.851 | 0.524-6.546 | 0.339 | 1.965 | 0.554-6.970 | 0.296 |
| Adjuvant therapy | No |  |  |  |  |  |  |  |  |  |  |  |  |
|  | CCRT | 0.425 | 0.094-1.915 | 0.265 | 0.467 | 0.104-2.096 | 0.320 | 0.497 | 0.111-2.231 | 0.361 | 0.532 | 0.119-2.382 | 0.409 |
|  | RT | 1.730 | 0.723-4.137 | 0.218 | 1.874 | 0.983-4.483 | 0.158 | 2.068 | 0.855-5.005 | 0.107 | 2.193 | 0.989-5.294 | 0.081 |
|  | CT | 0.751 | 0.497-1.134 | 0.174 | 0.779 | 0.516-1.177 | 0.236 | 0.834 | 0.554-1.256 | 0.384 | 0.859 | 0.571-1.294 | 0.468 |
| Histological grade | 1 |  |  |  |  |  |  |  |  |  |  |  |  |
|  | 2 | 0.743 | 0.433-1.272 | 0.279 | 0.727 | 0.425-1.242 | 0.243 | 0.974 | 0.580-1.635 | 0.921 | 0.966 | 0.576-1.619 | 0.895 |
|  | 3 | 1.061 | 0.564-1.998 | 0.854 | 1.106 | 0.588-2.081 | 0.756 | 1.317 | 0.704-2.462 | 0.389 | 1.367 | 0.732-2.550 | 0.326 |
| 7^th^ pathological stage | IB |  |  |  |  |  |  |  |  |  |  |  |  |
|  | IIA | 1.080 | 0.454-2.567 | 0.862 | 1.155 | 0.494-2.701 | 0.740 |  |  |  |  |  |  |
|  | IIB | 0.833 | 0.495-1.401 | 0.490 | 0.820 | 0.487-1.381 | 0.455 |  |  |  |  |  |  |
|  | IIIA | 1.661 | 0.935-2.949 | 0.084 | 1.675 | 0.949-2.953 | 0.075 |  |  |  |  |  |  |
|  | IIIB | 4.789 | 2.613-8.778 | < 0.001 | 4.835 | 2.659-8.794 | < 0.001 |  |  |  |  |  |  |
|  | IIIC | 4.632 | 2.537-8.458 | < 0.001 | 4.450 | 2.431-8.146 | < 0.001 |  |  |  |  |  |  |
| 8th pathological stage | IA |  |  |  |  |  |  |  |  |  |  |  |  |
|  | IB |  |  |  |  |  |  | 1.166 | 0.391-3.475 | 0.783 | 1.122 | 0.377-3.344 | 0.836 |
|  | IIA |  |  |  |  |  |  | 1.808 | 0.512-6.387 | 0.358 | 1.772 | 0.513-6.121 | 0.366 |
|  | IIB |  |  |  |  |  |  | 0.996 | 0.309-3.209 | 0.994 | 0.920 | 0.285-2.967 | 0.888 |
|  | IIIA |  |  |  |  |  |  | 1.196 | 0.324-4.424 | 0.788 | 1.074 | 0.289-3.991 | 0.915 |
|  | IIIB |  |  |  |  |  |  | 3.314 | 1.038-10.585 | 0.043 | 3.178 | 1.015-9.950 | 0.047 |
|  | IVA |  |  |  |  |  |  | 5.594 | 1.704-18.363 | 0.005 | 5.062 | 1.547-16.561 | 0.007 |
| SUVmax (median cutoff) | <4.7 |  |  |  |  |  |  |  |  |  |  |  |  |
|  | 4.7≤ | 1.577 | 1.034-2.406 | 0.034 |  |  |  | 1.458 | 0.929-2.287 | 0.101 |  |  |  |
| SUVmax  (best cutoff) | <3.4 |  |  |  |  |  |  |  |  |  |  |  |  |
|  | 3.4≤ |  |  |  | 1.669 | 1.009-2.763 | 0.046 |  |  |  | 1.637 | 0.987-2.715 | 0.056 |

**Supplementary Table 5. Univariate Cox regression analysis of survival in subjects with ^18^F-FDG PET/CT using discovery STE**

| **Variable** | **Categories** | **Disease-free survival** | | | | **Overall survival** | | | | |  |
| --- | --- | --- | --- | --- | --- | --- | --- | --- | --- | --- | --- |
|  |  | **Hazard ratio** | **95% confidence interval** | **P** | **P of log-rank test** | | **Hazard ratio** | **95% confidence interval** | **P** | **P of log-rank test** | |
| Sex | Female vs. Male | 4.161 | 0.578-29.970 | 0.157 | 0.1 | | 2.936 | 0.724-11.910 | 0.132 | 0.1 | |
| Age | <69 |  |  |  | 0.6 | |  |  |  | < 0.001 | |
|  | 69~78 | 1.309 | 0.750-2.282 | 0.343 |  |  | 1.140 | 0.675-1.926 | 0.625 |  |  |
|  | 78≤ | 1.142 | 0.627-2.080 | 0.664 |  |  | 2.373 | 1.471-3.827 | < 0.001 |  |  |
| Age  (1-yr increase) |  | 1.013 | 0.986-1.040 | 0.346 | 0.3 | | 1.039 | 1.015 | 1.064 | 0.001 | |
| Location | Cervical |  |  |  | 0.8 | |  |  |  | 0.8 | |
|  | Upper | 0.347 | 0.042-2.885 | 0.327 |  |  | 0.422 | 0.053-3.379 | 0.416 |  |  |
|  | Middle | 0.398 | 0.054-2.941 | 0.367 |  |  | 0.510 | 0.070-3.725 | 0.507 |  |  |
|  | Lower | 0.386 | 0.053-2.820 | 0.348 |  |  | 0.469 | 0.065-3.395 | 0.453 |  |  |
| Clinical N stage | N0 |  |  |  | < 0.001 | |  |  |  | < 0.001 | |
|  | N1 | 3.769 | 2.265-6.272 | < 0.001 |  |  | 2.177 | 1.431-3.312 | < 0.001 |  |  |
|  | N2 | 4.830 | 2.191-10.648 | < 0.001 |  |  | 2.400 | 1.175-4.902 | 0.016 |  |  |
|  | N3 | 42.465 | 9.690-186.108 | < 0.001 |  |  | 9.773 | 2.354-40.578 | 0.002 |  |  |
| Adjuvant therapy | No |  |  |  | 0.02 | |  |  |  | 0.08 | |
|  | CCRT | 2.954 | 0.407-21.461 | 0.284 |  |  | 2.396 | 0.331-17.340 | 0.387 |  |  |
|  | CT | 1.910 | 1.157-3.152 | 0.011 |  |  | 1.600 | 1.038-2.467 | 0.033 |  |  |
| Histological grade | 1 |  |  |  | 0.02 | |  |  |  | 0.4 | |
|  | 2 | 0.450 | 0.245-0.825 | 0.010 |  |  | 0.675 | 0.386-1.181 | 0.169 |  |  |
|  | 3 | 0.723 | 0.349-1.498 | 0.383 |  |  | 0.766 | 0.386-1.520 | 0.445 |  |  |
| 7^th^ pathological T stage | T1 |  |  |  | < 0.001 | |  |  |  | < 0.001 | |
|  | T2 | 1.683 | 0.762-3.717 | 0.198 |  |  | 2.252 | 1.263-4.018 | 0.006 |  |  |
|  | T3 | 6.153 | 3.694-10.250 | < 0.001 |  |  | 4.680 | 3.042-7.200 | < 0.001 |  |  |
|  | T4b | 7.042 | 0.954-51.980 | 0.056 |  |  | 2.789 | 0.383-20.308 | 0.311 |  |  |
| 8^th^ pathological T stage | T1a |  |  |  | < 0.001 | |  |  |  | < 0.001 | |
|  | T1b | 4.201 | 1.261-14.000 | 0.019 |  |  | 1.317 | 0.672-2.581 | 0.423 |  |  |
|  | T2 | 5.164 | 1.370-19.470 | 0.015 |  |  | 2.715 | 1.283-5.748 | 0.009 |  |  |
|  | T3 | 18.894 | 5.802-61.530 | < 0.001 |  |  | 5.642 | 2.967-10.731 | < 0.001 |  |  |
|  | T4b | 21.592 | 2.243-207.860 | 0.008 |  |  | 3.362 | 0.436-25.902 | 0.244 |  |  |
| Pathological N stage | N0 |  |  |  | < 0.001 | |  |  |  | < 0.001 | |
|  | N1 | 3.745 | 1.988-7.056 | < 0.001 |  |  | 1.951 | 1.184-3.214 | 0.009 |  |  |
|  | N2 | 6.884 | 3.664-12.932 | < 0.001 |  |  | 4.606 | 2.815-7.535 | < 0.001 |  |  |
|  | N3 | 15.467 | 6.627-36.098 | < 0.001 |  |  | 5.968 | 2.757-12.919 | < 0.001 |  |  |
| 7^th^ pathological substage | IB |  |  |  | < 0.001 | |  |  |  | < 0.001 | |
|  | IIB | 3.236 | 1.416-7.397 | 0.005 |  |  | 1.747 | 0.961-3.179 | 0.068 |  |  |
|  | IIIA | 10.220 | 4.670-22.378 | < 0.001 |  |  | 4.579 | 2.598-8.069 | < 0.001 |  |  |
|  | IIIB | 13.820 | 5.957-32.042 | < 0.001 |  |  | 8.778 | 4.676-16.479 | < 0.001 |  |  |
|  | IIIC | 18.450 | 7.089-48.037 | < 0.001 |  |  | 6.161 | 2.735-13.878 | < 0.001 |  |  |
| 8^th^ pathological substage | IB |  |  |  | < 0.001 | |  |  |  | < 0.001 | |
|  | IIA | 2.353 | 0.759-7.297 | 0.138 |  |  | 1.670 | 0.687-4.062 | 0.258 |  |  |
|  | IIB | 2.571 | 1.111-5.951 | 0.027 |  |  | 1.300 | 0.656-2.579 | 0.452 |  |  |
|  | IIIA | 3.758 | 1.478-9.556 | 0.005 |  |  | 2.199 | 1.029-4.701 | 0.042 |  |  |
|  | IIIB | 10.208 | 5.194-20.062 | < 0.001 |  |  | 5.891 | 3.570-9.722 | < 0.001 |  |  |
|  | IVA | 14.703 | 5.982-36.137 | < 0.001 |  |  | 5.543 | 2.502-12.280 | < 0.001 |  |  |
| SUVmax (median cutoff) | <4.7 |  |  |  | < 0.001 | |  |  |  | < 0.001 | |
|  | 4.7≤ | 6.261 | 3.530-11.100 | < 0.001 |  |  | 4.190 | 2.717-6.462 | < 0.001 |  |  |
| SUVmax (best cutoff) | <3.4 |  |  |  | < 0.001 | |  |  |  | <0.001 | |
|  | 3.4≤ | 7.538 | 3.607-15.750 | < 0.001 |  |  | 4.674 | 2.772-7.880 | < 0.001 |  |  |
| SUVmax (continuous) |  | 1.121 | 1.087-1.155 | < 0.001 | < 0.001 | | 1.079 | 1.054-1.105 | < 0.001 | < 0.001 | |

**Supplementary Table 6. Multivariate Cox regression analysis of disease-free survival in subjects with ^18^F-FDG PET/CT using discovery STE**

| **Variable** | **Categories** | **7^th^ staging,  SUVmax (median cutoff)** | | | **7^th^ staging,  SUVmax (best cutoff)** | | | **8^th^ staging,  SUVmax (median cutoff)** | | | **8^th^ staging,  SUVmax (best cutoff)** | | |
| --- | --- | --- | --- | --- | --- | --- | --- | --- | --- | --- | --- | --- | --- |
|  |  | **Hazard ratio** | **95% confidence interval** | **P** | **Hazard ratio** | **95% confidence interval** | **P** | **Hazard ratio** | **95% confidence interval** | **P** | **Hazard ratio** | **95% confidence interval** | **P** |
| Clinical  N stage | N0 |  |  |  |  |  |  |  |  |  |  |  |  |
|  | N1 | 1.673 | 0.946-2.960 | 0.077 | 1.574 | 0.888-2.789 | 0.121 | 1.666 | 0.944-2.941 | 0.078 | 1.594 | 0.905-2.805 | 0.106 |
|  | N2 | 1.085 | 0.446-2.638 | 0.858 | 1.088 | 0.448-2.643 | 0.852 | 1.162 | 0.485-2.782 | 0.737 | 1.158 | 0.485-2.764 | 0.741 |
|  | N3 | 19.826 | 3.999-98.301 | < 0.001 | 21.135 | 4.227-105.672 | < 0.001 | 19.273 | 3.682-100.872 | < 0.001 | 19.838 | 3.839-102.510 | < 0.001 |
| Adjuvant therapy | No |  |  |  |  |  |  |  |  |  |  |  |  |
|  | CCRT | 0.564 | 0.073-4.372 | 0.583 | 0.581 | 0.075-4.503 | 0.603 | 0.907 | 0.118-6.978 | 0.925 | 0.918 | 0.119-7.064 | 0.935 |
|  | CT | 0.599 | 0.333-1.077 | 0.087 | 0.584 | 0.324-1.052 | 0.073 | 0.667 | 0.365-1.219 | 0.188 | 0.651 | 0.357-1.185 | 0.160 |
| Histological grade | 1 |  |  |  |  |  |  |  |  |  |  |  |  |
|  | 2 | 0.825 | 0.397-1.715 | 0.607 | 0.788 | 0.379-1.638 | 0.523 | 0.688 | 0.355-1.332 | 0.267 | 0.676 | 0.351-1.304 | 0.243 |
|  | 3 | 1.012 | 0.430-2.384 | 0.998 | 0.936 | 0.395-2.219 | 0.881 | 0.892 | 0.411-1.936 | 0.773 | 0.850 | 0.392-1.840 | 0.680 |
| 7^th^ pathological stage | IB |  |  |  |  |  |  |  |  |  |  |  |  |
|  | IIB | 2.052 | 0.797-5.285 | 0.136 | 2.160 | 0.856-5.452 | 0.103 |  |  |  |  |  |  |
|  | IIIA | 6.000 | 2.227-16.167 | < 0.001 | 6.983 | 2.725-17.896 | < 0.001 |  |  |  |  |  |  |
|  | IIIB | 6.430 | 2.183-18.944 | < 0.001 | 7.787 | 2.818-21.519 | < 0.001 |  |  |  |  |  |  |
|  | IIIC | 10.470 | 2.878-38.082 | < 0.001 | 12.544 | 3.614-43.535 | < 0.001 |  |  |  |  |  |  |
| 8th pathological stage | IB |  |  |  |  |  |  |  |  |  |  |  |  |
|  | IIA |  |  |  |  |  |  | 1.277 | 0.380-4.286 | 0.693 | 1.226 | 0.374-4.026 | 0.737 |
|  | IIB |  |  |  |  |  |  | 1.992 | 0.761-5.215 | 0.161 | 1.969 | 0.760-5.100 | 0.163 |
|  | IIIA |  |  |  |  |  |  | 2.458 | 0.796-7.594 | 0.118 | 2.628 | 0.897-7.694 | 0.078 |
|  | IIIB |  |  |  |  |  |  | 5.219 | 2.080-13.099 | < 0.001 | 5.571 | 2.404-12.909 | < 0.001 |
|  | IVA |  |  |  |  |  |  | 8.136 | 2.366-27.983 | < 0.001 | 8.767 | 2.720-28.253 | < 0.001 |
| SUVmax (median cutoff) | <4.7 |  |  |  |  |  |  |  |  |  |  |  |  |
|  | 4.7≤ | 2.541 | 1.191-5.423 | 0.016 |  |  |  | 2.202 | 1.035-4.684 | 0.040 | 2.839 | 1.219-6.611 | 0.016 |
| SUVmax  (best cutoff) | <3.4 |  |  |  |  |  |  |  |  |  |  |  |  |
|  | 3.4≤ |  |  |  | 2.626 | 1.125-6.126 | 0.026 |  |  |  |  |  |  |

**Supplementary Table 7. Multivariate Cox regression analysis of overall survival in subjects with ^18^F-FDG PET/CT using discovery STE**

| **Variable** | **Categories** | **7^th^ staging,  SUVmax (median cutoff)** | | | **7^th^ staging,  SUVmax (best cutoff)** | | | **8^th^ staging,  SUVmax (median cutoff)** | | | **8^th^ staging,  SUVmax (best cutoff)** | | |
| --- | --- | --- | --- | --- | --- | --- | --- | --- | --- | --- | --- | --- | --- |
|  |  | **Hazard ratio** | **95% confidence interval** | **P** | **Hazard ratio** | **95% confidence interval** | **P** | **Hazard ratio** | **95% confidence interval** | **P** | **Hazard ratio** | **95% confidence interval** | **P** |
| Age | <69 |  |  |  |  |  |  |  |  |  |  |  |  |
|  | 69~78 | 1.094 | 0.623-1.924 | 0.754 | 1.064 | 0.605-1.874 | 0.829 | 0.958 | 0.546-1.680 | 0.880 | 0.903 | 0.513-1.590 | 0.724 |
|  | 78≤ | 2.310 | 1.314-4.063 | 0.004 | 2.385 | 1.364-4.169 | 0.002 | 2.149 | 1.270-3.637 | 0.004 | 2.093 | 1.239-3.536 | 0.006 |
| Clinical  N stage | N0 |  |  |  |  |  |  |  |  |  |  |  |  |
|  | N1 | 0.007 | 0.622-1.628 | 0.978 | 0.956 | 0.591-1.547 | 0.854 | 1.082 | 0.676-1.731 | 0.743 | 1.049 | 0.656-1.677 | 0.843 |
|  | N2 | 0.592 | 0.266-1.319 | 0.199 | 0.589 | 0.264-1.313 | 0.196 | 0.666 | 0.299-1.485 | 0.321 | 0.675 | 0.303-1.503 | 0.336 |
|  | N3 | 1.474 | 0.267-8.140 | 0.656 | 1.400 | 0.254-7.726 | 0.699 | 1.634 | 0.299-8.941 | 0.571 | 1.649 | 0.302-9.014 | 0.564 |
| 7^th^ pathological stage | IB |  |  |  |  |  |  |  |  |  |  |  |  |
|  | IIB | 1.171 | 0.606-2.261 | 0.639 | 1.255 | 0.665-2.368 | 0.484 |  |  |  |  |  |  |
|  | IIIA | 3.127 | 1.534-6.373 | 0.002 | 3.548 | 1.819-6.919 | < 0.001 |  |  |  |  |  |  |
|  | IIIB | 3.855 | 1.731-8.585 | < 0.001 | 4.534 | 2.146-9.583 | < 0.001 |  |  |  |  |  |  |
|  | IIIC | 3.800 | 1.263-11.435 | 0.018 | 4.504 | 1.568-12.938 | 0.005 |  |  |  |  |  |  |
| 8th pathological stage | IB |  |  |  |  |  |  |  |  |  |  |  |  |
|  | IIA |  |  |  |  |  |  | 1.128 | 0.433-2.939 | 0.806 | 1.180 | 0.468-2.974 | 0.726 |
|  | IIB |  |  |  |  |  |  | 1.063 | 0.521-2.171 | 0.866 | 1.100 | 0.543-2.227 | 0.792 |
|  | IIIA |  |  |  |  |  |  | 1.520 | 0.651-3.547 | 0.333 | 1.628 | 0.723-3.666 | 0.240 |
|  | IIIB |  |  |  |  |  |  | 3.525 | 1.795-6.924 | < 0.001 | 3.822 | 2.079-7.025 | < 0.001 |
|  | IVA |  |  |  |  |  |  | 3.597 | 1.221-10.597 | 0.020 | 3.846 | 1.370-10.799 | 0.011 |
| SUVmax (median cutoff) | <4.7 |  |  |  |  |  |  |  |  |  |  |  |  |
|  | 4.7≤ | 2.385 | 1.321-4.304 | 0.004 |  |  |  | 2.095 | 1.157-3.792 | 0.015 |  |  |  |
| SUVmax  (best cutoff) | <3.4 |  |  |  |  |  |  |  |  |  |  |  |  |
|  | 3.4≤ |  |  |  | 2.473 | 1.335-4.580 | 0.004 |  |  |  | 2.517 | 1.348-4.699 | 0.004 |
